# Total blood volume and red blood cell volume are not associated with orthostatic intolerance in adults with myalgic encephalomyelitis/chronic fatigue syndrome

**DOI:** 10.14814/phy2.71087

**Published:** 2026-09-09

**Authors:** Tadahiro Yamazaki, Donna M. Mancini, Michelle Blate, Patrick Quan, Anna Norweg, Dane B. Cook, Benjamin H. Natelson

**Affiliations:** ^1^ Department of Neurology Icahn School of Medicine at Mount Sinai New York New York USA; ^2^ Department of Cardiology Icahn School of Medicine at Mount Sinai New York New York USA; ^3^ Department of Population Health Science and Policy Icahn School of Medicine at Mount Sinai New York New York USA; ^4^ William S. Middleton Memorial Veterans Hospital Madison Wisconsin USA; ^5^ Department of Kinesiology University of Wisconsin–Madison Madison Wisconsin USA

**Keywords:** blood volume, lean test, myalgic encephalomyelitis/chronic fatigue syndrome, orthostatic intolerance, red blood cell volume

## Abstract

Orthostatic intolerance is common in myalgic encephalomyelitis/chronic fatigue syndrome (ME/CFS) and may worsen daily functioning. Although hypovolemia has been proposed as a contributor, its relationship to objective orthostatic abnormalities remains uncertain. We examined whether measured blood‐volume abnormalities were associated with standardized lean‐test outcomes. Adults meeting the 1994 ME/CFS criteria and reporting substantial post‐exertional malaise underwent a 10‐min lean test with capnography, followed 8 days later by total blood volume (TBV) measurement using the Daxor BVA‐100. Supine hypocapnia was defined as baseline end‐tidal CO_2_ (eTCO_2_) <34 mmHg; postural orthostatic syndrome of hypocapnia (POSH) as normal supine eTCO_2_ with any leaning value <34 mmHg; and postural orthostatic tachycardia syndrome (POTS) as a heart‐rate increase ≥30 beats/min or absolute heart rate ≥120 beats/min. Among 49 participants, TBV was hypovolemic in 35%, normovolemic in 43%, and hypervolemic in 22%; red blood cell volume was deficient in 49%. Overall, 55.1% had at least one lean‐test abnormality, most commonly POSH (40.8%). Abnormalities did not differ by TBV or red blood cell volume category. Static blood‐volume categories were not associated with lean‐test‐defined orthostatic abnormalities in ME/CFS, suggesting contributions from autonomic, vascular, respiratory, or cerebrovascular mechanisms.

## 1 INTRODUCTION

Myalgic encephalomyelitis/chronic fatigue syndrome (ME/CFS) is a debilitating chronic illness characterized by profound fatigue, post‐exertional malaise (PEM), unrefreshing sleep, cognitive dysfunction, and orthostatic intolerance (OI). Objective evaluation of OI commonly relies on measurement of heart rate and blood pressure during orthostatic challenge, including the 10‐min lean test (Natelson et al., 2022). Postural orthostatic tachycardia syndrome (POTS), defined by an excessive heart rate rise on standing, is a well‐recognized manifestation of OI in ME/CFS (Hoad et al., 2008; Natelson et al., 2022; van Campen et al., 2020). Orthostatic hypotension has also been reported (Bou‐Holaigah, 1995), although some studies have not found consistent differences from healthy controls (Jones et al., 2005; LaManca et al., 1999). More recently, lean‐test and tilt‐table studies have highlighted hypocapnic responses—either supine hypocapnia or postural orthostatic syndrome of hypocapnia (POSH)—as at least as frequent as POTS in ME/CFS (Naschitz et al., 2000, 2006; Natelson et al., 2007, 2022; Ocon et al., 2012).

A long‐standing hypothesis is that hypovolemia contributes to OI in ME/CFS (Streeten et al., 2000; Streeten & Scullard, 1996). Multiple studies have reported reduced total and/or compartmental blood volumes and smaller cardiac volumes or stroke volume in subsets of patients, supporting the biological plausibility of this mechanism (Hurwitz et al., 2009; Miwa & Fujita, 2008; Newton et al., 2016). These observations raise the possibility that reduced red blood cell mass and plasma volume may contribute to symptoms by limiting oxygen‐carrying capacity and effective circulating volume. One study suggested that patients with clinically suspected OI may have lower blood volumes than those without such symptoms (van Campen et al., 2018). However, orthostatic physiology and blood volume have not usually been assessed together; therefore, it remains unknown whether quantitatively defined hypovolemia is associated with physiologically defined OI during standardized testing. In addition, blood volume comprises functionally distinct components. Variation in red blood cell volume (RBCV)—from deficit to excess—could influence oxygen delivery, blood viscosity, and intravascular volume, as suggested in other chronic conditions (Miller et al., 2023), but whether such variation in RBCV modulates orthostatic responses in ME/CFS remains unknown.

We therefore examined whether quantitatively defined hypovolemia was associated with physiologically defined OI during a standardized 10‐min lean test in adults with ME/CFS. We further evaluated RBCV in addition to TBV to explore whether RBCV was related to OI. We hypothesized that OI would be more common in hypovolemic participants than in normovolemic or hypervolemic participants.

## 2 METHODS

### 2.1 Ethics approval

The study was approved by the Mount Sinai Institutional Review Board (STUDY‑20‑02014); all participants provided written informed consent. All procedures were conducted in accordance with the principles of the World Medical Association Declaration of Helsinki.

### 2.2 Study design and participants

This study was a cross‐sectional analysis of prospectively collected pre‐intervention data from a prospective, single‐center NIH‐funded parent study designed to determine the relationship between performance on two successive cardiopulmonary exercise tests (CPETs) and PEM, the sine qua non of ME/CFS. The parent study was prospectively registered on ClinicalTrials.gov (NCT04740736) before participant enrollment began. The study was conducted at the Pain & Fatigue Study Center, Department of Neurology, Icahn School of Medicine at Mount Sinai (New York, USA), between August 2021 and July 2025. Adults aged 25–60 years fulfilled the 1994 case definition for ME/CFS (Fukuda et al., 1994). To ensure a substantial current symptom burden at intake, participants were additionally required to report a score of ≥3 on a 0–5 Likert scale for at least three of the following seven non‐PEM symptoms: sore throat; tender cervical or axillary lymph nodes; headache; myalgia; arthralgia; problems with attention or concentration; and unrefreshing sleep. They were also required to report a score of ≥3 for PEM. Exclusion criteria were (i) any medical condition that could explain the fatigue, including anemia identified by routine laboratory testing, (ii) current treatment with medications known to blunt cardiovascular responses to exertion, and (iii) a structured psychiatric interview documenting psychotic disorders, bipolar disorder, anorexia nervosa, or bulimia nervosa within 5 years, alcohol or drug abuse within 2 years, or current major depressive disorder. All participants completed a 10‐min lean test 8 days before the quantitative blood volume assessment, and both assessments were completed before CPETs. Sex was recorded as female or male based on participant self‐report at enrollment.

### 2.3 Ten‐minute lean test

A standardized 10‐min lean test, originally developed by NASA to detect spaceflight‐related orthostatic intolerance, was administered to all participants (Bungo et al., 1985). After a minimum 10‐min supine rest, baseline physiological parameters—blood pressure, heart rate, respiratory rate, and end‐tidal CO_2_ (eTCO_2_), measured with a portable capnograph/pulse oximeter (Nellcor N‐85 Microstream, Covidien LLC, Mansfield, MA, USA)—were recorded in triplicate at 1‐min intervals. Participants then stood with their feet together approximately 6–8 inches from a wall and leaned against the wall without moving for 10 min, during which the same parameters were measured at 1‐min intervals.

### 2.4 Blood volume assessment

We used the semiautomated Daxor BVA‐100 system to measure intravascular volumes. This system provides determinations of TBV, plasma volume (PV), and RBCV within approximately 60 min with high accuracy (Manzone et al., 2007). After collection of a 5 mL baseline blood sample, human serum albumin labeled with ^131^I was injected intravenously over 1 min and allowed to equilibrate. Additional 5 mL blood samples were drawn at 15, 21, 27, 33, and 39 min to correct for albumin transudation. Plasma volume at time zero was extrapolated from at least three samples with a coefficient of variation ≤3.9%, and TBV was calculated as TBV = PV + RBCV. All volumes were normalized to each participant's ideal body size to account for interindividual differences (Feldschuh & Enson, 1977). Established blood volume analysis interpretive ranges use different thresholds for TBV and RBCV because RBCV varies more among healthy individuals: TBV is interpreted in 8% increments, whereas RBCV is interpreted in 10% increments (Manzone et al., 2007). Accordingly, TBV was categorized as hypovolemia (<−8% deviation), normovolemia (−8% to +8%), or hypervolemia (>+8%) in accordance with prior ME/CFS blood‐volume data (Hurwitz et al., 2009). RBCV deficit was defined as an RBCV more than 10% below the ideal predicted volume, normal RBCV as an RBCV within ±10% of ideal, and RBCV excess as an RBCV more than 10% above ideal (Miller et al., 2023). An RBCV deficit indicates reduced circulating red cell mass by BVA and, when routine hemoglobin or hematocrit values are normal, may represent occult or otherwise unrecognized anemia (Miller & Mullan, 2015).

### 2.5 Definitions of orthostatic outcomes

Supine hypocapnia was diagnosed when any baseline eTCO_2_ measurement was <34 mmHg. POSH was defined as all supine eTCO_2_ values ≥34 mmHg with at least one leaning eTCO_2_ value <34 mmHg. POTS was defined as a ≥30 beats/min increase in heart rate or an absolute heart rate ≥120 beats/min at any time during leaning. “Any lean‐test abnormality” denoted the presence of at least one of these three findings (supine hypocapnia, POSH, or POTS); “no lean‐test abnormality” indicated that none occurred. In our protocol, supine hypocapnia was treated as a resting abnormality identified during the orthostatic testing sequence, based on prior work linking low supine eTCO_2_ to orthostatic intolerance (Natelson et al., 2007).

### 2.6 Statistical analysis

Normality of continuous variables was assessed with the Shapiro–Wilk test. Non‐normal data are summarized as median [interquartile range (IQR)]. Group comparisons across the three TBV categories (hypovolemic, normovolemic, and hypervolemic) were performed with the Kruskal–Wallis test for continuous variables and Fisher's exact test for categorical variables. Similarly, analyses across the three RBCV categories (deficit, normal, and excess) used the Kruskal–Wallis test or Fisher's exact test. A two‐sided *p* < 0.05 was considered statistically significant. Analyses were performed in R 4.4.3 (R Foundation for Statistical Computing, Vienna, Austria).

## 3 RESULTS

### 3.1 Participant characteristics

Forty‐nine participants with ME/CFS (40 women and 9 men) completed both the lean test and blood volume analysis. The median age was 36 years [29–46]. Seventeen participants (35%) were classified as hypovolemic, 21 (43%) as normovolemic, and 11 (22%) as hypervolemic. Age and sex did not differ significantly across the three TBV groups (Table 1). Twenty‐four participants (49%) had an RBCV deficit, 20 (41%) had normal RBCV, and 5 (10%) had RBCV excess. Age and sex did not differ significantly across the three RBCV groups (Table 2). The RBCV deficits indicated reduced circulating red cell mass by BVA and were compatible with occult anemia if routine hemoglobin and hematocrit values were normal.

**TABLE 1:** Baseline characteristics stratified by total blood volume status.

	Total (*n* = 49)	Hypovolemic (*n* = 17)	Normovolemic (*n* = 21)	Hypervolemic (*n* = 11)	*p*
Age, years (median [IQR])	36 [29–46]	36 [31–45]	34 [29–47]	36 [29–41.5]	0.59^a^
Female, *n* (%)	40 (81.6%)	14 (82.4%)	17 (81.0%)	9 (81.8%)	1.00^b^

*Note*: Age distribution was non‐normal (Shapiro–Wilk test, *p* < 0.01); values are therefore shown as median [IQR].

Abbreviation: IQR, interquartile range.

^a^ Kruskal–Wallis test.

^b^ Fisher's exact test.

**TABLE 2:** Baseline characteristics stratified by red blood cell volume status.

	Total (*n* = 49)	Deficit (*n* = 24)	Normal (*n* = 20)	Excess (*n* = 5)	*p*
Age, years (median [IQR])	36 [29–46]	37.5 [30–49.75]	31 [29–51]	45 [38–46]	0.066^a^
Female, *n* (%)	40 (81.6%)	19 (79.2%)	17 (85.0%)	4 (80.0%)	0.88^b^

*Note*: Age distribution was non‐normal (Shapiro–Wilk test, *p* < 0.01); values are therefore shown as median [IQR].

Abbreviations: IQR, interquartile range.

^a^ Kruskal–Wallis test.

^b^ Fisher's exact test.

### 3.2 Lean‐test outcomes

Twenty‐seven participants (55.1%) had at least one lean‐test abnormality: supine hypocapnia in 6 (12.2%), POSH in 20 (40.8%), POTS in 6 (12.2%), and both POSH and POTS in 4 (8.2%). The frequencies of orthostatic outcomes did not differ significantly across the TBV groups (Table 3). Consistent with the TBV findings, RBCV status showed no significant association with any lean‐test outcome (Table 4). There were also no significant sex differences in any orthostatic outcome (Table 5).

**TABLE 3:** Lean‐test findings by total blood volume status across all subjects.

	Total (*n* = 49)	Hypovolemic (*n* = 17)	Normovolemic (*n* = 21)	Hypervolemic (*n* = 11)	*p*
Supine hypocapnia	6 (12.2%)	2 (11.8%)	2 (9.5%)	2 (18.2%)	0.86
POSH	20 (40.8%)	8 (47.1%)	9 (42.9%)	3 (27.3%)	0.65
POTS	6 (12.2%)	2 (11.8%)	4 (19.0%)	0 (0.0%)	0.40
POSH + POTS	4 (8.2%)	1 (5.9%)	3 (14.3%)	0 (0.0%)	0.53
Any lean test abnormality	27 (55.1%)	11 (64.7%)	11 (52.4%)	5 (45.5%)	0.58

*Note*: Fisher's exact test was performed in all analyses.

Abbreviations: POSH, postural orthostatic syndrome of hypocapnia; POTS, postural orthostatic tachycardia syndrome.

**TABLE 4:** Lean‐test findings by red blood cell volume status across all subjects.

	Total (*n* = 49)	Deficit (*n* = 24)	Normal (*n* = 20)	Excess (*n* = 5)	*p*
Supine hypocapnia	6 (12.2%)	2 (8.3%)	4 (20.0%)	0 (0.0%)	0.40
POSH	20 (40.8%)	12 (50.0%)	5 (25.0%)	3 (60.0%)	0.20
POTS	6 (12.2%)	3 (12.5%)	3 (15.0%)	0 (0.0%)	1.00
POSH + POTS	4 (8.2%)	2 (8.3%)	2 (10.0%)	0 (0.0%)	1.00
Any lean test abnormality	27 (55.1%)	15 (62.5%)	9 (45.0%)	3 (60.0%)	0.50

*Note*: Fisher's exact test was performed in all analyses.

Abbreviations: POSH, postural orthostatic syndrome of hypocapnia; POTS, postural orthostatic tachycardia syndrome.

**TABLE 5:** Sex distribution across orthostatic intolerance subgroups.

	Total (*n* = 49)	Female (*n* = 40)	Male (*n* = 9)	*p*
Supine hypocapnia	6 (12.2%)	6 (15.0%)	0 (0.0%)	0.58
POSH	20 (40.8%)	18 (45.0%)	2 (22.2%)	0.28
POTS	6 (12.2%)	5 (12.5%)	1 (11.1%)	1.00
POSH + POTS	4 (8.2%)	4 (10.0%)	0 (0.0%)	1.00
Any lean test abnormality	27 (55.1%)	24 (60.0%)	3 (33.3%)	0.27

*Note*: Fisher's exact test was performed in all analyses.

Abbreviations: POSH, postural orthostatic syndrome of hypocapnia; POTS, postural orthostatic tachycardia syndrome.

## 4 DISCUSSION

In this single‐center cohort of adults with ME/CFS, quantitatively measured TBV category was not associated with orthostatic outcomes on a standardized 10‐min lean test, including supine hypocapnia, POSH, or POTS. More than half of participants exhibited at least one orthostatic abnormality, yet the distribution of these abnormalities was similar across hypovolemic, normovolemic, and hypervolemic groups. These findings suggest that static blood volume deficits alone are unlikely to be the principal determinant of OI in ME/CFS. Identifying hypovolemia may still be clinically relevant because volume expansion is a logical therapeutic target in this subgroup. However, randomized trials of fludrocortisone in ME/CFS have shown limited benefit (Rowe et al., 2001), implying that correction of hypovolemia alone is often insufficient and that additional mechanisms must be addressed. Our results extend prior work by showing that, even when blood volume is measured quantitatively, TBV category does not distinguish specific physiological OI subtypes.

One study that explicitly compared blood volume between ME/CFS subgroups defined by the presence or absence of clinically suspected OI is the pilot study by van Campen et al. (van Campen et al., 2018). Using a dual‐isotope technique, they found lower absolute blood volumes and greater deficits from predicted values among patients with day‐to‐day symptoms consistent with OI than among those without such symptoms. In their cohort, clinical suspicion of OI was based on history, and detailed orthostatic physiology was not collected contemporaneously with blood volume assessment. By combining quantitative blood volume analysis with a standardized lean test incorporating capnography, our study suggests that static blood volume status is insufficient to account for the spectrum of orthostatic patterns observed during standardized testing.

We also examined RBCV categories because reduced red cell mass could plausibly impair oxygen delivery and provoke compensatory tachycardia or hyperventilation, whereas RBCV excess could influence blood viscosity and intravascular volume. An RBCV deficit detected by BVA represents a direct reduction in circulating red cell mass; if peripheral hemoglobin and hematocrit values are normal, this phenotype can be considered an occult red‐cell mass deficit rather than conventional CBC‐defined anemia (Miller & Mullan, 2015). In other orthostatic intolerance cohorts, low RBCV has been associated with orthostatic symptoms, excessive orthostatic reductions in cerebral blood flow velocity, and POTS (Carew et al., 2009; Lin et al., 2014; Raj et al., 2005), supporting biological plausibility. However, neither RBCV deficit nor RBCV excess was associated with any lean‐test outcome in our cohort. These null findings suggest that, at the level of static compartmental volumes, variation in red cell mass does not independently determine orthostatic responses in ME/CFS. The small number of participants with RBCV excess, however, limited our ability to detect modest effects of extreme RBCV phenotypes, and dynamic measures of oxygen delivery and extraction during orthostatic stress may be necessary to reveal more subtle contributions of RBCV to symptom generation.

Multiple mechanisms beyond absolute blood volume likely contribute to OI in ME/CFS. Impaired venous constriction and increased venous capacitance can promote gravitational pooling and reduce venous return (Streeten & Scullard, 1996). Autonomic dysregulation, with reduced vagal modulation and altered heart rate variability, has been demonstrated at rest, during sleep, and during orthostatic challenge (Boneva et al., 2007; Sisto et al., 1995). Upright posture can substantially reduce cerebral blood flow even when heart rate and blood pressure remain within conventional limits (van Campen et al., 2020). Neurohumoral and neural abnormalities, including a renin–aldosterone mismatch and small‐fiber neuropathy in subsets of patients with POTS, may further impair cardiovascular control (Gibbons et al., 2013; Raj et al., 2005). Finally, deconditioning can reduce plasma volume and ventricular mass over weeks (Levine et al., 1997), but deconditioning alone is unlikely to explain the high prevalence of OI in ME/CFS; in a controlled ME/CFS study, cerebral blood flow reduction during tilt was independent of deconditioning as defined by CPET, and patients had greater cerebral blood flow reduction than healthy controls (van Campen et al., 2021). Together, these observations are consistent with our finding that TBV category alone did not determine lean‐test orthostatic outcomes and underscore the need to integrate autonomic, vascular, respiratory, and cerebral perfusion assessments when characterizing orthostatic subtypes in ME/CFS.

This study has several limitations. The modest sample size reduced power for subgroup analyses, and recruitment from a single tertiary care center of participants well enough to undergo testing may underrepresent more severely affected patients. We used the 1994 Fukuda case definition (Fukuda et al., 1994) to maintain comparability with our previous cohorts, while requiring a substantial current symptom burden at intake, with PEM mandatory rather than optional. Our center also participated in a CDC‐led study of seven U.S. ME/CFS specialty clinics that found few statistically significant and no clinically meaningful between‐clinic differences in standardized measures of ME/CFS symptoms and function, supporting the clinical comparability of patients evaluated at our center with those evaluated at other expert centers (Unger et al., 2024). Nevertheless, replication of the present findings in cohorts recruited explicitly using contemporary case definitions, including the 2015 Institute of Medicine criteria (Committee on the Diagnostic Criteria for Myalgic Encephalomyelitis/Chronic Fatigue Syndrome et al., 2015), is warranted. The absence of a healthy control group precluded direct comparison of blood‐volume patterns and orthostatic responses between individuals with ME/CFS and healthy volunteers. The 8‐day interval between the lean test and blood volume assessment, together with the lack of standardization of acute hydration and salt intake, may have introduced temporal variability in blood volume, eTCO_2_, and hemodynamic measures. We did not assess state anxiety during lean testing or analyze respiratory tidal volume; therefore, anxiety‐related hyperventilation and other breathing‐pattern abnormalities cannot be excluded as contributors to supine hypocapnia or POSH. We used predicted ideal volumes to define hypovolemia, which may have led to misclassification in some individuals. Finally, the 10‐min lean test imposes milder orthostatic stress than head‐up tilt; hypovolemia‐driven hemodynamic instability might manifest preferentially during more prolonged or more severe orthostatic challenges.

In conclusion, OI was highly prevalent in this ME/CFS cohort, but static hypovolemia did not appear to account for its occurrence. Within the limitations of this single‐center study, neither TBV nor RBCV category was associated with lean‐test‐defined orthostatic abnormalities, including supine hypocapnia, POSH, or POTS. Comprehensive pathophysiological models that integrate blood volume status with autonomic, vascular, respiratory, and cerebral perfusion factors will be essential for the development of rational, phenotype‐directed therapies. Multicenter studies incorporating dynamic measurements of cerebral blood flow, respiratory pattern, and CO_2_ regulation during graded orthostatic stress are needed to advance this agenda.

## AUTHOR CONTRIBUTIONS

**Tadahiro Yamazaki:** Conceptualization; data curation; formal analysis; methodology; visualization. **Donna M. Mancini:** Conceptualization; funding acquisition; investigation; project administration; resources; supervision. **Michelle Blate:** Data curation; investigation. **Patrick Quan:** Data curation; investigation. **Anna Norweg:** Project administration; supervision. **Dane B. Cook:** Funding acquisition; methodology. **Benjamin H. Natelson:** Conceptualization; funding acquisition; resources; supervision.

## FUNDING INFORMATION

This work was supported by the National Institutes of Health [R01 NS117638 to Benjamin H. Natelson and Donna M. Mancini] and the Department of Veterans Affairs Career Scientist Award [#2IK6RD002734‐06 to Dane B. Cook]. The funders had no role in the study design, data collection, data analysis, data interpretation, writing of the manuscript, or decision to submit the article for publication.

## CONFLICT OF INTEREST STATEMENT

The authors declare that there are no competing interests associated with the manuscript.

## ETHICS STATEMENT

The study was approved by the Mount Sinai Institutional Review Board on April 5, 2021 (approval number: STUDY‐20‐02014). All procedures were performed in accordance with the World Medical Association Declaration of Helsinki, relevant laws, and institutional guidelines. The privacy rights of participants were observed.

## CONSENT TO PARTICIPATE

All participants provided written informed consent before participation.

## Data Availability

De‐identified data supporting the findings of this study are not publicly available because they contain sensitive human‐subject data and are subject to participant privacy and ethical restrictions. Reasonable requests may be considered by the corresponding author with appropriate ethical approvals and data‐use agreements.
